# Learning Together During a Pandemic Lockdown: Connecting Older Mentors with Nursing Students

**DOI:** 10.1093/geroni/igaa057.3478

**Published:** 2020-12-16

**Authors:** Alison Phinney, Frances Affleck

**Affiliations:** 1 University Of British Columbia, Vancouver, British Columbia, Canada; 2 University of British Columbia, Vancouver, British Columbia, Canada

## Abstract

Nursing education tends to focus on complex clinical issues affecting older adults who are acutely ill or in long-term care. This creates challenges for educators wanting to expose students to a greater range of experience, including realities of healthy aging. Opportunities to do things differently were presented when an established undergraduate nursing course on complex aging care underwent significant adjustment in the early months of the COVID-19 pandemic. As the course was condensed and moved online and clinical sites closed, invitations were extended to community-dwelling older people who wanted to “help teach nursing students about aging”. The response was overwhelming; over nine days, 118 people (ages 65-94) volunteered to be mentors. Through weekly online/ phone conversations, each person guided their assigned student to learn about diverse experiences of aging. Post-survey results showed the impact of these conversations. Over 90% of mentors felt they had contributed in a meaningful way to student learning and would do it again and recommend it to others. 85% of students felt it was a meaningful experience, offering comments like: “I am more mindful of my assumptions now” and “I learned to approach interactions with older adults as a collaboration; we have so much to give each other”. These results provide a needed counterpoint to the predominant COVID discourse of older people as “isolated, helpless, and needy”. Students came to understand that older people were also “engaged, active, and contributing” and identified how this had changed their view of aging. Implications for nursing education are explored.

